# How Does Work Motivation Impact Employees’ Investment at Work and Their Job Engagement? A Moderated-Moderation Perspective Through an International Lens

**DOI:** 10.3389/fpsyg.2020.00038

**Published:** 2020-02-21

**Authors:** Or Shkoler, Takuma Kimura

**Affiliations:** ^1^Independent Researcher, Netanya, Israel; ^2^Graduate School of Career Studies, Hosei University, Tokyo, Japan

**Keywords:** intrinsic and extrinsic motivation, heavy work investment, job engagement, work status, moderated moderation, cultural differences

## Abstract

This paper aims at shedding light on the effects that intrinsic and extrinsic motivation, as predictors, have on heavy work investment of time and effort and on job engagement. Using a questionnaire survey, this study conducted a moderated-moderation analysis, considering two conditional effects—worker’s status (working students vs. non-student employees) and country (Israel vs. Japan)—as potential moderators, since there are clear cultural differences between these countries. Data were gathered from 242 Israeli and 171 Japanese participants. The analyses revealed that worker’s status moderates the effects of intrinsic and extrinsic motivation on heavy work investment of time and effort and on job engagement and that the moderating effects were conditioned by country differences. Theoretical and practical implications and future research suggestions are discussed.

## Introduction

Our world today has been described by the acronym VUCA (volatile, uncertain, complex, and ambiguous). In this rapidly changing world, organizations and individuals need to engage in continuous learning. To achieve a competitive advantage, organizations need to develop organizational learning, which can be achieved by acquiring learning individuals. From the latter’s viewpoint, it is getting more necessary for workers to learn continuously to enhance and maintain their employability. As shown in previous research, the number of people engaging in lifelong learning has significantly increased (Corrales-Herrero and Rodríguez-Prado, 2018).

In such an era, an organization needs to acquire and retain learning individuals. However, it is not an easy task because they might have turnover intentions, even when they are motivated to work. Since learning individuals enhance their skills continuously and have a “third place” for new encounters (e.g., school), they are likely to find other attractive job opportunities. Therefore, it is valuable for us to explore how motivation affects learning individuals’ attitudes and behavior. However, to the best of our knowledge, researchers have not addressed this issue.

Recently, researchers and practitioners have paid increasing attention to employees’ job engagement (JE) (Bailey et al., 2017). Previous studies suggested that engaged workers are likely to achieve high performance and have low intention to leave (Rich et al., 2010; Alarcon and Edwards, 2011). However, JE does not necessarily represent workers’ favorable attitude (van Beek et al., 2011). In the case of working individuals, their appearance of being “highly engaged” can be caused by time constraints or impression management motive.

Recognizing the ambiguous nature of “engaged workers,” this study also focuses on a relatively new construct called heavy work investment (HWI). People high in HWI are apparently similar to those high in JE. However, as will be discussed later, these two constructs are distinct. By focusing on both engagement and HWI, we can reveal the underlying mechanism of how motivation affects the learning individuals’ engagement.

To address these issues, we analyzed quantitative data which include both learning individuals (hereafter called “working students”) and non-student workers. The choice of employees who are students as opposed to “regular” employees was based on arguments presented in the conservation of resources (COR) theory (Hobfoll, 1989, 2011). It will be elaborated further in this paper.

Besides, since the contexts of lifelong learning and work in an organization can affect the focal mechanism, we collected data from two countries—Israel and Japan—and conducted a between-country comparative analysis. As we will discuss below, these two countries widely differ in cultural dimensions, as suggested by Hofstede (1980, 2018). We limit the scope of the research to Israel and Japan to concentrate on a specific issue which was not investigated in previous studies, especially in a comparison between these two countries (to the best of our knowledge). The sample and analysis of this study can provide insightful implications because these two countries are widely different in their national cultural contexts.

### Work Motivation

A general definition of motivation is the psychological force that generates complex processes of goal-directed thoughts and behaviors. These processes revolve around an individual’s internal psychological forces alongside external environmental/contextual forces and determine the direction, intensity, and persistence of personal behavior aimed at a specific goal(s) (Kanfer, 2009; Kanfer et al., 2017). In the work domain, work motivation is “a set of energetic forces that originate within individuals, as well as in their environment, to initiate work-related behaviors and to determine their form, direction, intensity and duration” (after Pinder, 2008, p. 11). As mentioned, work motivation is derived from an interaction between individual differences and their environment (e.g., cultural, societal, and work organizational) (Latham and Pinder, 2005). In addition, motivation is affected by personality traits, needs, and even work fit, while generating various outcomes and attitudes, such as satisfaction, organizational citizenship behaviors (OCBs), engagement, and more (for further reading, see Tziner et al., 2012).

Moreover, work motivation, as an umbrella term under the self-determination theory (SDT), is usually broken down into two main constructs—intrinsic versus extrinsic motivation (Ryan and Deci, 2000b). On the one hand, intrinsic motivation is an internal driver. Employees work out of the excitement, feeling of accomplishment, joy, and personal satisfaction they derive both from the processes of work-related activities and from their results (Deci and Ryan, 1985; Bauer et al., 2016; Legault, 2016). On the other hand, extrinsic motivation maintains that the individual’s drive to work is influenced by the organization, the work itself, and the employee’s environment. These can range from social norms, peer influence, financial needs, promises of reward, and more. As such, being extrinsically motivated is being focused on the utility of the activity rather than the activity itself (see Deci and Ryan, 1985; Legault, 2016). However, this does not, by any means, point that extrinsic motivation is less effective than intrinsic motivation (Deci et al., 1999).

Furthermore, the SDT (Ryan and Deci, 2000b) argues that each type of motivation is on opposite poles of a single continuum. However, we agree with the notion that they are mutually independent, as Rockmann and Ballinger (2017) wrote:

“…there is increasing evidence that intrinsic and extrinsic motivations are independent, each with unique antecedents and outcomes … in organizations, because financial incentives exist alongside interesting tasks, individuals can simultaneously experience extrinsic and intrinsic motivation for doing their work.” (p. 11)

Literature-wise, the intrinsic–extrinsic outlook of motivation lacks coherent research, and to the best of our knowledge, most of the past research addressed the intrinsic part (e.g., Rich et al., 2010; Bauer et al., 2016). As such, we would align with the approach to distinguish the two work motivations as was reviewed in this section and consequently treat it as a predictor in our research.

### Job Engagement

Work engagement is typically defined as “a positive, fulfilling, work-related state of mind that is characterized by vigor, dedication, and absorption” (Schaufeli et al., 2002, p. 74). As such, engaged employees appear to be hardworking (*vigor*), are more involved in their work (*dedication*), and are more immersed in their work (*absorption*) (see also Bakker et al., 2008; Chughtai and Buckley, 2011; Taris et al., 2015). JE was initially proposed as a positive construct (Kahn, 1990), and empirical studies revealed that a high level of JE leads to positive work outcomes. For example, recent studies exhibited its positive effect on individual job performance and adverse effect on turnover intention (Breevaart et al., 2016; Owens et al., 2016; Shahpouri et al., 2016; Kumar et al., 2018). Therefore, employees’ JE has been regarded as one of the performance indicators of human resource management.

In terms of antecedents and predictors, it is broadly accepted that JE may be affected by both individual differences (e.g., Sharoni et al., 2015; Latta and Fait, 2016; Basit, 2017) and environmental/contextual elements (e.g., Sharoni et al., 2015; Basit, 2017; Gyu Park et al., 2017; Lebron et al., 2018) (see also Macey and Schneider, 2008) or even an interaction between these two factors (e.g., Sharoni et al., 2015; Hernandez and Guarana, 2018).

### Intrinsic/Extrinsic Motivation and JE

To the best of our knowledge, only a few papers examined the association between work motivation and JE. For instance, Rich et al. (2010) tested a model in which both intrinsic motivation and JE were tested “vertically,” meaning they were both mediators (in the model) rather than two factors in a predictor–outcome relationship. This offers a further incentive to examine the association between (intrinsic/extrinsic) work motivation and JE.

Because JE is “…driven by perceptions of psychological meaningfulness, safety, and availability at work” (Hernandez and Guarana, 2018, p. 1), a vital notion behind work motivation is the perception of the job as a place for fulfilling different needs: extrinsic needs, such as income and status, and intrinsic needs, such as enjoyment, and personal challenge. This perception, very likely, bolsters the association between the employees’ drive to work and the workplace or the work themselves, increasing the involvement and the amount of work they put into their work (i.e., JE). These assumptions lead us to hypothesize the following:

*H1*: Intrinsic motivation positively associates with JE.

*H2*: Extrinsic motivation positively associates with JE.

### Heavy Work Investment

Fundamentally different from being immersed or involved at work (e.g., JE), employees usually invest time and energy at their workplace with various manifestations, which ultimately barrel down to the concept of HWI. This umbrella term encompasses two major core aspects: (1) investment of time (i.e., working long hours) and (2) investment of effort and energy (i.e., devoting substantial efforts, both physical and mental, at work) (Snir and Harpaz, 2012, Snir and Harpaz, 2015). These dimensions are, respectively, called (a) time commitment (HWI-TC) and work intensity (HWI-WI). Notably, many studies deal with the implications of working overtime (e.g., Stimpfel et al., 2012; Caruso, 2014). However, to the best of our knowledge, empirical studies regarding the investment of efforts at work as an indicator of HWI (e.g., Tziner et al., 2019) are scarce. Therefore, the current research addresses both of the core dimensions of HWI (i.e., *time* [HWI-TC] and *effort* [HWI-WI]).

In reality, HWI consists of many different constructs (e.g., workaholism and work addiction or passion to work) but conclusively revolves around the devotion of time and effort at work (see Snir and Harpaz, 2015, p. 6). HWI is apparently similar to JE, but these two constructs are distinct. As shown in previous studies, the correlation between workaholism—one component of HWI—and JE is generally weak, and engaged individuals can be not only high in HWI but also low in HWI (van Beek et al., 2011).

For HWI’s possible predictors, Snir and Harpaz (2012, 2015) have differentiated between situational and dispositional types of HWI (based on Weiner’s, 1985, attributional framework). Examples of situational types are financial needs or employer-directed contingencies (external factors), while dispositional types are characterized by individual differences (internal factors), such as work motivation.

### Intrinsic/Extrinsic Motivation and HWI

As previously mentioned, employees may be driven to work by both intrinsic and extrinsic forces, motivating them to engage in work activities to fulfill different needs (e.g., salary, enjoyment, challenge, and promotion). Ultimately, these two mutually exclusive elements would translate into the same outcome—increased investment at work. At this juncture, however, we cannot say what type of work motivation (intrinsic/extrinsic) would more tightly link to either (1) the heavier devotion of time (HWI-TC) or (2) the heavier investment of effort (HWI-WI) at work. Consequently, we hypothesize further the following:

*H3*: Intrinsic motivation positively associates with both HWI-TC and HWI-WI.

*H4*: Extrinsic motivation positively associates with both HWI-TC and HWI-WI.

### HWI and JE

It is important to emphasize that, again, HWI and JE are mutually independent constructs. Nevertheless, HWI points at two different investment “types”—in time and effort. Theoretically, we see that although both aspects of investment are, probably, linked to JE, we may also conclude that these associations would differ based on the type of investment. For example, while workers may allegedly spend a great deal of time on the job, in actuality, they may not be working (studiously) on their given tasks at all, a situation labeled as “presenteeism” (see Rabenu and Aharoni-Goldenberg, 2017). However, exerting more effort at work, by definition, means that one is more engaged, to whatever extent, in work (e.g., investing more effort, basically, means investing time as well, but *not* vice versa). In other words, while we expect that JE will be positively related to dimensions of HWI (one must devote time and invest more effort to be engaged at work), we also assume that JE will be more strongly correlated with the *effort* dimension, rather than *time*. As such, we hypothesize the following:

*H5a*: JE positively associates with HWI-TC.

*H5b*: JE positively associates with HWI-WI.

*H5c*: JE has a stronger association with HWI-WI than with HWI-TC.

The purpose of H5a–H5c is to differentiate JE from HWI-WI and HWI-TC, as they may have some overlaps. However, they are still stand-alone constructs, which is the reason the current research gauge them both and correlate them, though they are both outcome variables (an issue of convergent and discriminant validity).

### Worker’s Status—Buffering Effect

An organization or a workplace is usually composed of several types of employees, albeit not all of them exhibit the same attitudes and behaviors at work. For example, temporary workers report greater job insecurity and lower well-being than permanent employees (Dawson et al., 2017). Another example is of students (i.e., working students vs. non-student employees). The motivators and incentives needed to drive corporate/working students differ from others. They are, for instance, more interested in salary, promotion, tangible rewards in their job, and other such benefits (Palloff and Pratt, 2003).

Furthermore, capitalizing upon the COR theory (Hobfoll, 1989, 2011), the main argument is that employees invest various resources (e.g., time, energy, money, effort, and social credibility) at work. The more resources devoted, the less will remain at the individual’s disposal, and prolonged state of depleted resources without gaining others may result in stress and, ultimately, burnout. As such, a worker who is also a student will, by definition, have fewer resources at either domain (work, social life, or family), as opposed to a worker who does not engage in any form of higher education at all. Working students are under severer time constraints than non-student employees because they face “work–study conflict.” Therefore, compared to non-student workers, working students have difficulty in devoting so much time and physical as well as psychological effort to work. Specifically, working students with a low level of motivation may take an interest in studies and thus not be likely to devote much effort to work. However, motivated working students will maintain their effort through effective time management because they highly value their current work. Thus, JE and HWI of working students will depend on their motivation to a greater degree than non-student workers. Ergo, we posit that the associations between intrinsic/extrinsic motivation and HWI and JE are conditioned by the type of worker under investigation.

For the current study, the notion of working students versus non-student employees would be gauged, as not much attention was given to distinguishing both groups in research. Usually, samples were composed of either group distinctively, not in tandem with one another. Hence, we hypothesize the following, based on our previous hypotheses:

*H6*: Worker’s status moderates the relationship between intrinsic motivation and HWI-TC, HWI-WI, and JE, such that the relationship will be weaker for working students than for non-student employees.

*H7*: Worker’s status moderates the relationship between extrinsic motivation and HWI-TC, HWI-WI, and JE, such that the relationship will be weaker for working students than for non-student employees.

### Country Difference—Buffering Effect

Worker’s status’ moderation of the links between intrinsic/extrinsic motivation to HWI and JE, as mentioned above, does not appear in a vacuum. This conditioning may also be dependent on international cultural differences. That is to say, we assume that we would receive different results based on the country under investigation because the social, work, cultural, and national values differ from one country to another. Firstly, culture, in this sense, may be defined as “common patterns of beliefs, assumptions, values, and norms of behavior of human groups (represented by societies, institutions, and organizations)” (Aycan et al., 2000, p. 194). As mentioned, countries differ from one another in many aspects. The most prominent example is the cultural/national dimensions devised by Hofstede (1980, 1991). Different countries display different cultural codes, norms, and behaviors, which may affect their market and work values and behaviors. As such, it is safe to assume that work-related norms and codes differ from one country to another to the extent that working students may exhibit or express certain attitudes and behaviors in country X, but different ones in country Y. The same goes for non-student (or “regular”) workers, as well.

In this study, we examine the case of Israel’s versus Japan’s different situation and cultural perspectives in the work sense. Japan’s culture is more hierarchical and formal than the Israeli counterpart. Japanese believe efforts and hard work may bring “anything” (e.g., prosperity, health, and happiness), while in Israel, there is much informal communication, and “respect” is earned by (hands-on) experience, not necessarily by a top-down hierarchy. Japanese emphasize loyalty, cohesion, and teamwork (Deshpandé et al., 1993; Deshpandé and Farley, 1999). Compared to Israeli, Japanese employees are more strongly required to conform to the organization’s norm and dedicate themselves to the organization’s future. Such cultural characteristics may affect the working attitudes and behavior of working students. Specifically, in Japan, working students try to devote as much time as possible even if they are under severe time constraints caused by the study burden. Moreover, sometimes, they experience guilt because they use their time for themselves (i.e., study) rather than for the firm (e.g., socializing with colleagues). Thus, they engage in much overtime work as a tactic of impression management (Leary and Kowalski, 1990) to make themselves look loyal and hard working.

In addition, in Israel, there is high value to performance, while in Japan, competition (between groups, usually) is rooted in society and drives for excellence and perfection. Also, Israelis respect tradition and normative cognition. They tend to “live the present,” rather than save for the future, while Japanese people tend to invest more (e.g., R&D) for the future. Even in economically difficult periods, Japanese people prioritize steady growth and own capitals rather than short-term revenues such that “companies are not here to make money every quarter for the shareholders, but to serve the stakeholders and society at large for many generations to come” (for further reading, see Hofstede, 2018).

In Hofstede’s use of the term, some aspects of these cultural differences can be summarized as Japan being higher in power distance, masculinity, and long-term orientation than Israel (Hofstede, 2018). These cultural differences led us to formulate the following hypotheses:

*H8*: Country differences condition the moderation of worker’s status on the relationship between intrinsic motivation and HWI-TC, HWI-WI, and JE, such that the effect of worker’s status suggested in H6 will be weaker for Japanese than for Israelis.

*H9*: Country differences condition the moderation of worker’s status on the relationship between extrinsic motivation and HWI-TC, HWI-WI, and JE, such that the effect of worker’s status suggested in H7 will be weaker for Japanese than for Israelis.

It is important to note, however, that H8 and H9 are also developed to increase the external validity of the research and its generalizability beyond a single culture, as Barrett and Bass (1976) noted that “most research in industrial and organizational psychology is done within one cultural context. This context puts constraints upon both our theories and our practical solutions to the organizational problem” (p. 1675).

**FIGURE 1:**
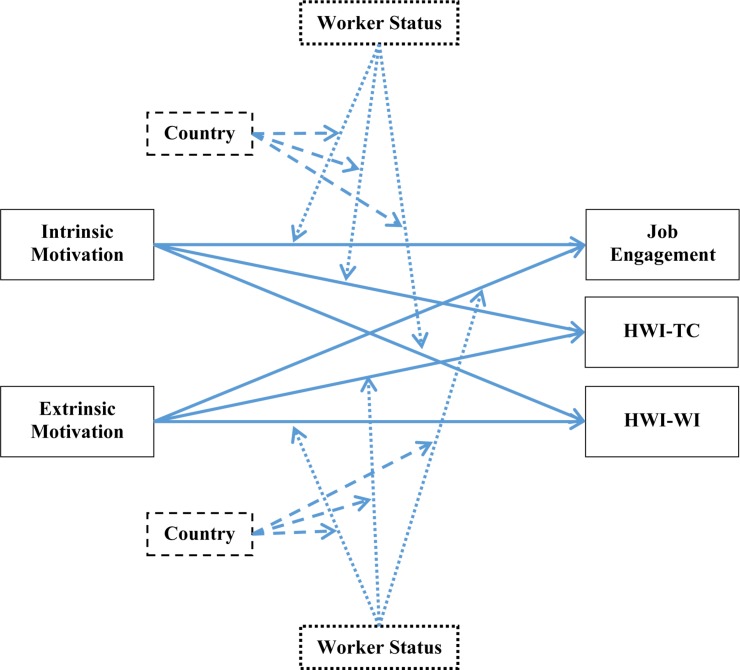
Research model. Worker’s status: 1 = working students, 2 = non-student employees. Country: 1 = Israel, 2 = Japan. HWI-TC = time commitment dimension of heavy work investment. HWI-WI = work intensity dimension of heavy work investment.

Figure 1 portrays the overall model.

## Materials and Methods

For hypothesis testing, this study conducted questionnaire-based research using samples of company employees who also engage in a manner of higher education (i.e., working students) and those who do not (i.e., “regular” or non-student employees). Since working students in both countries do not concentrate in specific age groups, industries, or functional areas, participants were recruited from various fields. Moreover, to reduce the impact of organization-specific culture, we collected data from various companies rather than from a specific company, in both countries.

### Participants

The research constitutes 242 Israeli (70.9% response rate) and 171 Japanese (56.6% response rate) participants, from various industries and organizations. The demographical and descriptive statistics for each sample are presented in Table 1. The table also contains the result of group difference tests, pointing at some demographic differences between Israeli and Japanese samples. Therefore, the following analyses include these demographics as control variables to control their potential influence on the research model and reduce the problem that would arise from said differences between the two countries.

**TABLE 1:** Demographical and descriptive statistics for the Israeli (*N* = 242) and the Japanese (*N* = 171; in parenthesis) samples.

**Parameter**	**Category**	**%**	**Range**	***M***	***SD***	**Diff. test^1^**
Gender	Female	36.8 (49.7)	–	–	–	χ^2^ = 2.55
	Male	63.2 (50.3)	–	–	–	
Marital status	Single	49.2 (31.0)	–	–	–	χ^2^ = 5.31
	Married	45.9 (64.3)	–	–	–	
	Divorced	5.0 (4.7)	–	–	–	
Job position	Non-managerial	74.0 (42.7)	–	–	–	χ^2^ = 6.70**
	Managerial	26.0 (57.3)	–	–	–	
Worker status	Working student	31.8 (56.7)	–	–	–	χ^2^ = 3.99*
	Regular employees	68.2 (43.3)	–	–	–	
Age	–	–	22–55 (24–70)	35.26 (45.57)	9.95 (8.93)	*t* = 10.81***
No. of children	–	–	0–6 (0–4)	1.47 (0.87)	1.75 (1.02)	*t* = 4.03**
Tenure	–	–	0.5–19 (1–40)	5.60 (12.38)	4.99 (9.80)	*t* = 9.21***

*p < 0.05, **p < 0.01, ***p < 0.001. ^1^Diff. test = difference test to compare Israel and Japan as groups in the demographical data.

### Measures

The items of the questionnaire were initially written in English and then translated into Hebrew and Japanese, utilizing the back-translation procedure (Brislin, 1980).

*Work motivation* was gauged by the Work Extrinsic and Intrinsic Motivation Scale (WEIMS; Tremblay et al., 2009), consisting of 18 Likert-type items ranging from 1 (“Does not correspond at all”) to 6 (“Corresponds exactly”). *Intrinsic motivation* had a high reliability (α_*Israel*_ = 0.92, α_*Japan*_ = 0.86; e.g., “…Because I derive much pleasure from learning new things”) as did *extrinsic motivation* (α_*Israel*_ = 0.73, α_*Japan*_ = 0.75; e.g., “…For the income it provides me”).

HWI (see Snir and Harpaz, 2012) was tapped by 10 Likert-type items ranging from 1 (“Strongly disagree”) to 6 (“Strongly agree”), five items for each dimension, namely, *time commitment* (HWI-TC; e.g., “Few of my peers/colleagues put in more weekly hours to work than I do”) and *work intensity* (HWI-WI; e.g., “When I work, I really exert myself to the fullest”), respectively. *HWI-TC* had a high reliability (α_*Israel*_ = 0.85, α_*Japan*_ = 0.92) as did *HWI-WI* (α_*Israel*_ = 0.95, α_*Japan*_ = 0.91).

*JE* was gauged by the Utrecht Work Engagement Scale-9 (UWES-9; Schaufeli et al., 2006) consisting of nine Likert-type items ranging from 1 (“Strongly disagree”) to 6 (“Strongly agree”). The measure had a very high reliability (α_*Israel*_ = 0.95, α_*Japan*_ = 0.94; e.g., “I am immersed in my work”).

### Procedure

For the *Israeli* sample, a pencil-and-paper research survey was distributed to 341 total potential participants in two universities and one college. One of the authors provided the questionnaire in several courses (MBA and management, human resource management, psychology, and more), at the end of each class session. Those wishing to participate replied affirmatively and were included in the total sample. We assured the anonymity and discretion of the participants and the data derived from the research and included a conscious consent question at the beginning of the survey asking for their agreement to participate. No incentives were given whatsoever to the participants for their cooperation. A total of 341 surveys were distributed, yet only 242 came back filled, and all of them were valid to use as data in the research.

For the *Japanese* sample, the data were collected by using the online questionnaire system of Google spreadsheet. Invitation messages were sent to the potential respondents via email or SNS messenger with the link of the questionnaire. One of the authors contacted 189 full-time workers who participated in one or more of the following (1) strategic management and organization management classes of a Japanese private university, (2) human resource management course in an educational service company, or (3) one-off lectures conducted by the author. All of them were non-student workers, and ultimately, 97 of them answered the questionnaire in full (51.3% response rate). As for the working students, the same author reached out to three graduate schools through personal networks. Then, he asked the liaison of each school to list up working students and send them the questionnaire link by email or SNS messenger. In total, the link was sent to 113 working students (in said three universities), and 74 completed the questionnaire (65.5% response rate). Thus, the overall response rate was 56.6%.

### Data Analyses

The data were analyzed utilizing the SPSS (v. 23) software package and PROCESS macro for SPSS (v. 3.3). PROCESS is an add-on macro for the SPSS and SAS software packages written by Andrew F. Hayes. It is a modeling tool based on ordinary least squares (OLS) and logistic regressions for basic and complex path analyses with strong algorithms and modular capabilities and can handle simultaneous moderation and mediations effects (including moderated-moderation effects).

The choice of PROCESS (over SEM) is based on methodological and mathematical reasons. To elaborate, holistic testing of the entire model (see Figure 1) via SEM will result in 15 different observed variables (including the interaction effects) and a two-group comparison, and abundant regression lines would result in a high number of degrees of freedom. It would also require a considerably higher sample size to meet the mathematical conditions for SEM. However, we should note that one of the limitations of PROCESS is the inability to test models with more than one dependent variable (*Y*) or more than one independent variable (*X*), and as such it is required to test the model (see Figure 1) separately—one for each predictor–criterion linkage.

### Control Variables

As per Table 1, we can see some differences between the two countries, and as such, we included them as covariates in the moderated-moderation analyses. In other words, in these analyses, we controlled for the effects of job position, age, number of children, tenure, and also gender and marital status. This is relevant for Tables 4–6. Evidently, the inclusion of control variables has increased the predictive capacity and goodness of our results. *Gender* is a dichotomous closed question with options of (1) male or (2) female. *Age* is an open question: “what is your age (in years)? ______.” *Marital status* is a closed question with options of (1) single, (2) married, (3) divorced, or (4) widowed. *Number of children* is an open question: “How many children do you have? ______.” *Tenure* is an open question: “what is your tenure at work (in years)? ______.” *Job position* is a dichotomous closed question with options of (1) non-managerial or (2) managerial.

### Common Method Bias

Harman’s one-factor test (Podsakoff et al., 2003) was used to assess the degree to which intercorrelations among the variables might be an artifact of common method variance (CMV). The first general factor that emerged from the analysis accounted only for 35.19% of the explained variance in the Israeli sample and 37.27% in the Japanese sample. While this result does not rule out completely the possibility of same-source bias (CMV), according to Podsakoff et al. (2003), less than 50% of the explained variance accounted for by the first emerging factor indicates that CMV is an unlikely explanation of our investigation findings.

## Results

First, we explored descriptive statistics and associations between the variables. These results are displayed in Tables 2, 3, for each sample.

**TABLE 2:** Pearson correlation matrix for working students (*below* the diagonal; *n* = 77) and non-student employees (*above* the diagonal; *n* = 165), means and standard deviations in the Israeli sample (*N* = 242).

	**1**	**2**	**3**	**4**	**5**	***M*_*ws*_ (*M*_*nse*_)**	***SD*_*we*_ (*SD*_*nse*_)**
(1) Intrinsic motivation	–	0.87	0.39	0.29	0.59	4.50 (3.98)	0.90 (0.84)
(2) Extrinsic motivation	0.87	–	0.36	0.38	0.74	4.27 (3.94)	1.48 (1.36)
(3) HWI-TC	0.78	0.85	–	0.33	0.30	3.85 (4.44)	1.48 (1.00)
(4) HWI-WI	0.47	0.73	0.69	–	0.77	4.77 (5.07)	1.66 (1.03)
(5) Job engagement	0.76	0.88	0.55	0.76	–	4.25 (4.04)	1.72 (1.28)

*All correlations are significant at *p* < 0.001. HWI-TC, time commitment dimension of heavy work investment. HWI-WI, work intensity dimension of heavy work investment. An indication of *ws* in the mean and standard deviation columns = working students’ group. An indication of *nse* in the mean and standard deviation columns = non-student employees’ group.*

**TABLE 3:** Pearson correlation matrix for working students (*below* the diagonal; *n* = 97) and non-student employees (*above* the diagonal; *n* = 74), means and standard deviations in the Japanese sample (*N* = 171).

	**1**	**2**	**3**	**4**	**5**	***M*_*ws*_ (*M*_*nse*_)**	***SD*_*we*_ (*SD*_*nse*_)**
(1) Intrinsic motivation	–	0.69	0.36	0.48	0.60	3.26 (3.45)	0.72 (0.81)
(2) Extrinsic motivation	0.65	–	0.38	0.58	0.81	4.00 (4.20)	1.09 (1.14)
(3) HWI-TC	**0.14**	**0.12**	–	0.50	0.30	2.47 (2.50)	1.36 (1.36)
(4) HWI-WI	0.46	0.55	0.50	–	0.62	3.79 (3.96)	1.09 (1.17)
(5) Job engagement	0.45	0.81	**0.15**	0.71	–	3.99 (4.10)	1.04 (1.06)

*All correlations are significant at *p* < 0.001, apart from bolded correlations, which are non-significant (*p* > 0.05). HWI-TC, time commitment dimension of heavy work investment. HWI-WI, work intensity dimension of heavy work investment. An indication of *ws* in the mean and standard deviation columns = working students’ group. An indication of *nse* in the mean and standard deviation columns = non-student employees’ group.*

As shown in Table 2, we found the following regarding the Israeli sample:

- JE positively correlates with HWI-TC for working students, *r*(77) = 0.55, *p* = 0.000, and for non-student employees *r*(165) = 0.30, *p* = 0.000 (supporting H5a, in Israel).
- JE positively correlates with HWI-WI for working students, *r*(77) = 0.76, *p* = 0.000, and for non-student employees *r*(165) = 0.77, *p* = 0.000 (supporting H5b, in Israel).

These differences in correlation coefficients are in line with our H5c, meaning JE has stronger links to HWI-WI as opposed to HWI-TC. Ergo, in order to gauge whether these differences are statistically significant, we used Fisher’s *Z* transformation and significance test. For working students, the difference is indeed significant (*Z* = 2.31, *p* = 0.021) and is also for the non-student employees’ group (*Z* = 6.41, *p* = 0.000). This supports H5c, in Israel.

Moreover, as shown in Table 3, we found the following regarding the Japanese sample:

- JE positively correlates with HWI-TC *only* for non-student employees, *r*(74) = 0.30, *p* = 0.001, but is non-significant for working students, *r*(94) = 0.15, *p* = 0.146 (partially supporting H5a, in Japan).
- JE positively correlates with HWI-WI for working students, *r*(94) = 0.72, *p* = 0.000, and for non-student employees, *r*(74) = 0.62, *p* = 0.000 (supporting H5b, in Japan).

These differences in correlation coefficients are in line with our H5c, meaning JE has stronger links to HWI-WI as opposed to HWI-TC. Ergo, in order to gauge whether these differences are statistically significant, we used Fisher’s *Z* transformation and significance test. For working students, the difference is indeed significant (*Z* = 5.12, *p* = 0.000) and is also significant for the non-student employees’ group (*Z* = 2.48, *p* = 0.013). This supports H5c, in Japan.

To test the rest of our hypotheses (i.e., H1–H4 and H6–H9), we utilized the PROCESS macro for SPSS using model no. 3 for moderated moderation (95% bias-corrected bootstrapping with 5,000 resamples). The results from the analyses are presented in Tables 4–6. However, it is important to note that we also used heteroscedasticity-consistent standard error (SE) estimators, as suggested by Hayes and Cai (2007), to ensure that the estimator of the covariance matrix of the parameter estimates will not be biased and inconsistent under heteroscedasticity violation.

**TABLE 4:** Moderficients and confidence intervals (CIs) for predicting HWI-TC.

**Predictors**	**HWI-TC**		**Predictors**	**HWI-TC**	
	***b***	**95% CI^1^**		***b***	**95% CI**
**Dependent varialbes**					
I-Mot	4.26	[3.16, 5.36]***	E-Mot	2.98	[2.19, 3.78]***
Worker status^2^	10.11	[7.35, 12.88]***	Worker status	7.39	[5.14, 9.63]***
Country^3^	8.99	[5.73, 12.24]***	Country	5.78	[3.17, 8.38]***
INT_1_ (Mot × Status)	–1.99	[−2.66, −1.34]***	INT_1_	–1.47	[−1.97, −0.98]***
INT_2_ (Mot × Country)	–2.18	[−3.05, −1.29]***	INT_2_	–1.56	[−2.16, −0.96]***
INT_3_ (Status × Country)	–5.64	[−7.53, −3.75]***	INT_3_	–4.26	[−5.85, −2.67]***
INT_4_ (Mot × Status × Country)	1.18	[0.66, 1.70]***	INT_4_	0.87	[0.50, 1.26]***
**Control variables**					
Gender	0.09	[−0.14, 0.32]		0.06	[−0.17, 0.29]
Age	0.01	[−0.01, 0.03]		0.01	[−0.01, 0.02]
Marital status	0.15	[−0.12, 0.41]		0.18	[−0.08, 0.44]
Number of children	–0.03	[−0.14, 0.09]		0.01	[−0.10, 0.11]
Tenure	–0.02	[−0.04, −0.01]*		–0.02	[−0.04, −0.01]*
Job position	0.08	[−0.18, 0.35]		0.08	[−0.17, 0.34]

*p < 0.05, ***p < 0.001. DV, dependent variable. HWI-TC, time commitment dimension of heavy work investment. Mot, motivation. I-Mot, intrinsic motivation. E-Mot, extrinsic motivation. INT, interaction effect. ^1^95% CI with 5,000 resampling via bias-corrected bootstrapping. ^2^Worker status (1 = working student, 2 = non-student employee). ^3^Country (1 = Israel, 2 = Japan).

**TABLE 5:** Moderated-moderation regression coefficients and confidence intervals (CIs) for predicting HWI-WI.

**Predictors**	***HWI-WI***			***HWI-WI***	
	***b***	**95% CI^1^**		***b***	**95% CI**
**Dependent variables**					
I-Mot	1.33	[0.19, 4.73]*	E-Mot	1.35	[0.63, 2.06]***
Worker status^2^	4.69	[0.56, 8.82]*	Worker status	4.60	[2.50, 6.70]***
Country^3^	2.56	[−2.07, 6.79]	Country	1.89	[−0.45, 4.24]
INT_1_ (Mot × Status)	–0.67	[−1.56, 0.21]	INT_1_	–0.72	[−1.14, −0.29]**
INT_2_ (Mot × Country)	–0.33	[−1.37, 0.71]	INT_2_	–0.38	[−0.88, 0.11]
INT_3_ (Status × Country)	–2.32	[−4.74, 0.10]^♢^	INT_3_	–2.25	[−3.66, −0.84]**
INT_4_ (Mot × Status × Country)	0.34	[−0.23, 0.91]	INT_4_	0.36	[0.06, 0.67]*
**Control variables**					
Gender	0.01	[0.19, 0.21]		–0.04	[−0.23, 0.14]
Age	–0.01	[−0.02, 0.01]		–0.01	[−0.02, 0.01]
Marital status	–0.20	[−0.43, 0.03]		–0.23	[−0.43, −0.02]*
Number of children	–0.13	[−0.22, −0.03]*		–0.09	[−0.18, −0.01]*
Tenure	–0.01	[−0.03, −0.01]		–0.01	[−0.02, 0.01]
Job position	0.27	[0.04, 0.50]*		0.21	[0.01, 0.42]*

^♢^*p* < 0.01, *p < 0.05, **p < 0.01, ***p < 0.001. DV, dependent variable. HWI-WI, work intensity dimension of heavy work investment. Mot, motivation. I-Mot, intrinsic motivation. E-Mot, extrinsic motivation. INT, interaction effect. ^1^95% CI with 5,000 resampling via bias-corrected bootstrapping. ^2^Worker status (1 = working student, 2 = non-student employee). ^3^Country (1 = Israel, 2 = Japan).

**TABLE 6:** Moderated-moderation regression coefficients and confidence intervals (CIs) for predicting job engagement (JE).

**Predictors**	**JE**		**Predictors**	**JE**	
	***b***	**95% CI^1^**		***b***	**95% CI**
**Dependent variables**					
I-Mot	3.53	[2.21, 4.86]***	E-Mot	1.95	[1.41, 2.50]***
Worker status^2^	7.20	[3.79, 10.60]***	Worker status	3.90	[2.12, 5.68]***
Country^3^	7.72	[4.30, 11.42]***	Country	2.82	[1.10, 4.55]**
INT_1_ (Mot × Status)	–1.43	[−2.19, −0.68]***	INT_1_	–0.78	[−1.14, −0.42]***
INT_2_ (Mot × Country)	–1.50	[−2.39, −0.62]***	INT_2_	–0.54	[−0.90, −0.17]**
INT_3_ (Status × Country)	–3.81	[−5.94, −1.69]***	INT_3_	–1.82	[−2.97, −0.68]**
INT_4_ (Mot × Status × Country)	0.78	[0.26, 1.30]**	INT_4_	0.36	[0.12, 0.60]**
**Control variables**					
Gender	0.20	[−0.01, 0.40]		0.07	[−0.09, 0.23]
Age	–0.01	[−0.03, 0.01]		–0.02	[−0.03, −0.01]**
Marital status	–0.17	[−0.41, 0.06]		–0.22	[−0.40, −0.04]*
Number of children	0.01	[−0.11, 0.09]		0.05	[−0.02, 0.13]
Tenure	–0.01	[−0.03, 0.01]		–0.01	[−0.03, −0.01]*
Job position	0.42	[0.18, 0.65]***		0.33	[0.16, 0.51]***

*p < 0.05, **p < 0.01, ***p < 0.001. DV, dependent variable. JE, job engagement. Mot, motivation. I-Mot, intrinsic motivation. E-Mot, extrinsic motivation. INT, interaction effect. ^1^95% CI with 5,000 resampling via bias-corrected bootstrapping. ^2^Worker status (1 = working student, 2 = non-student employee). ^3^Country (1 = Israel, 2 = Japan).

**FIGURE 2:**
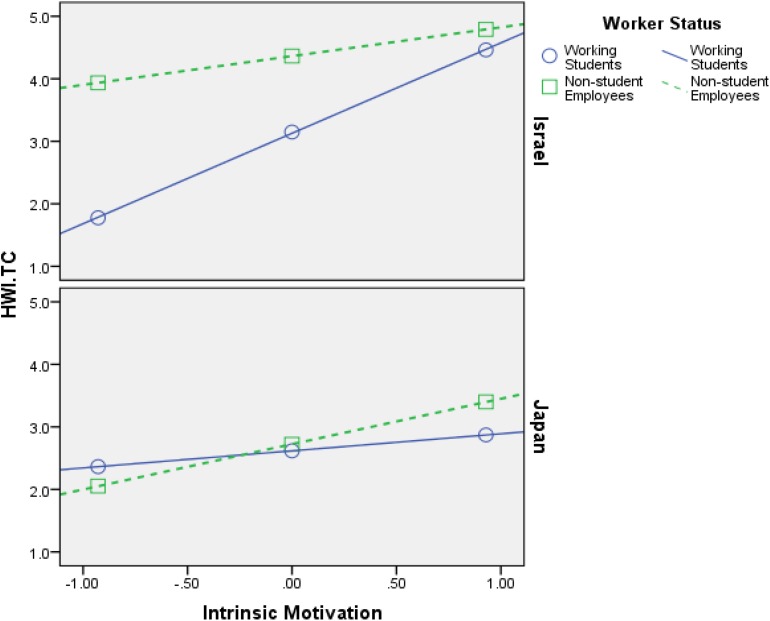
Interaction effects of Intrinsic Motivation × Worker’s Status × Country in predicting HWI-TC. HWI-TC, time commitment dimension of heavy work investment.

**FIGURE 3:**
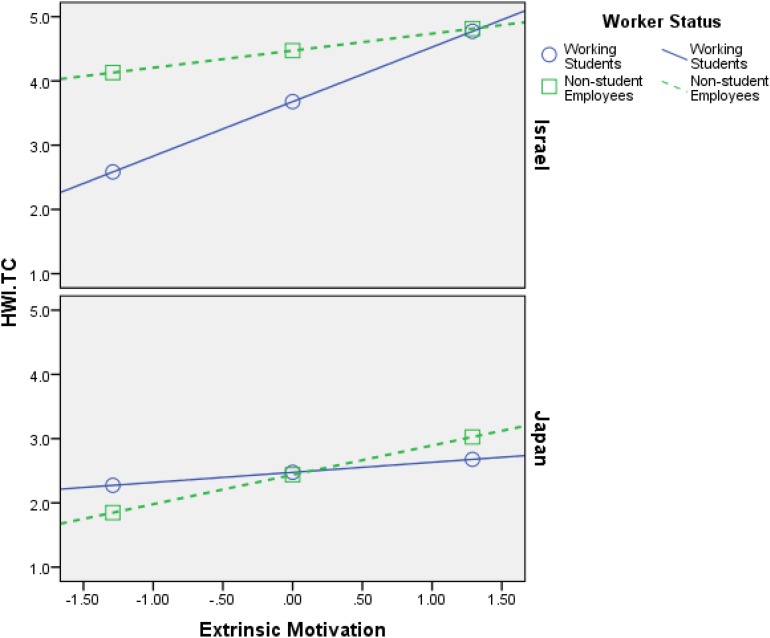
Interaction effects of Extrinsic Motivation × Worker’s Status × Country in predicting HWI-TC. HWI-TC, time commitment dimension of heavy work investment.

**FIGURE 4:**
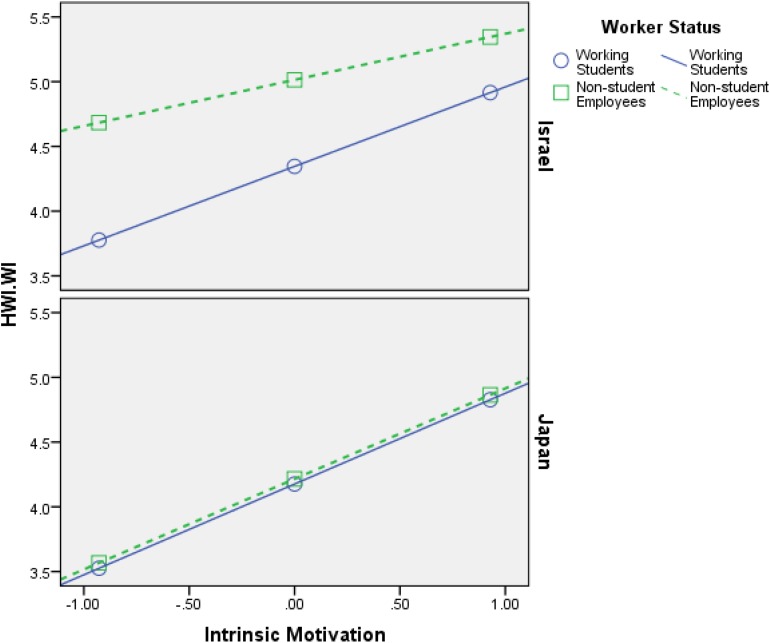
Interaction effects of Intrinsic Motivation × Worker’s Status × Country in predicting HWI-WI. HWI-WI, work intensity dimension of heavy work investment.

**FIGURE 5:**
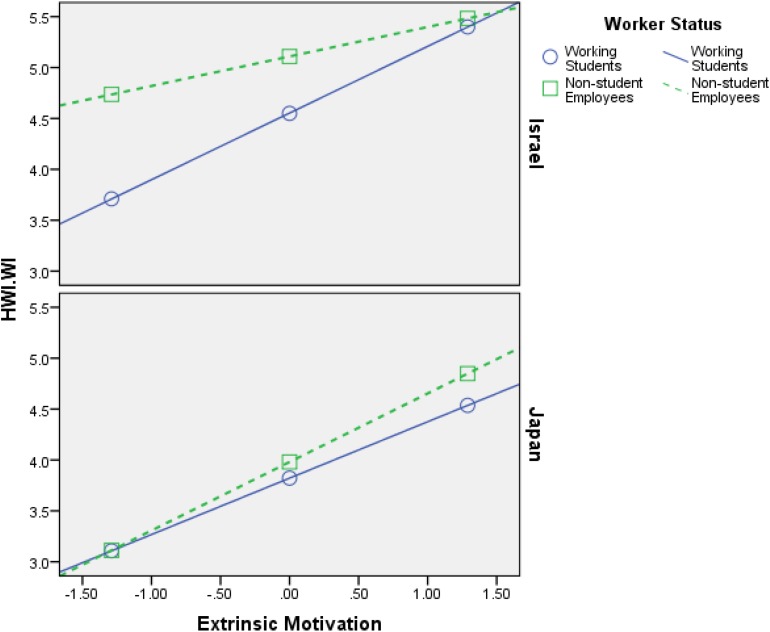
Interaction effects of Extrinsic Motivation × Worker’s Status × Country in predicting HWI-WI. *Notes*. HWI-WI = work intensity dimension of heavy work investment.

**FIGURE 6:**
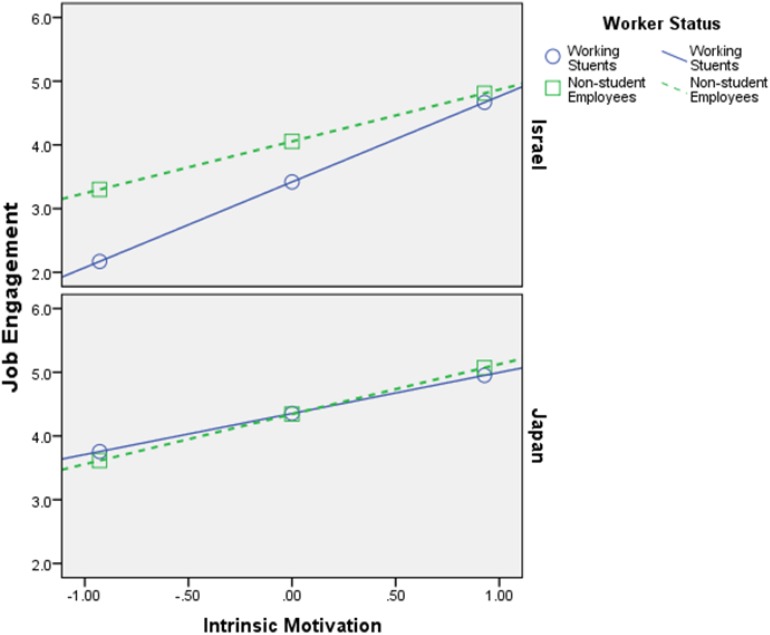
Interaction effects of Intrinsic Motivation × Worker’s Status × Country in predicting job engagement.

**FIGURE 7:**
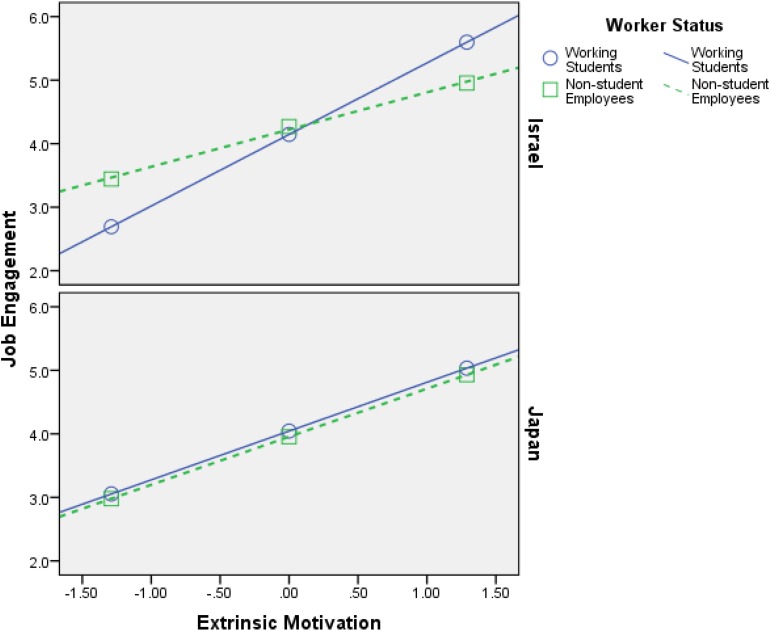
Interaction effects of Extrinsic Motivation × Worker’s Status × Country in predicting job engagement.

Firstly, the findings that are shown in Tables 4–6 support **H1**–**H4**, meaning both intrinsic motivation and extrinsic motivation relate positively to HWI-TC, HWI-WI, and JE, in all samples (Israel and Japan). Additionally, the interaction effects (most of them) are significant, which is the most important part of any moderation analysis (see Appendix in Shkoler et al., 2017). Figures 2–7 portray moderation effects.

Figures 2–7 display surprising findings:

(1) The behaviors of the correlations (for instance, between intrinsic motivation and JE or HWI-TC) are different between the two countries, in general, such that means and correlations are both higher in the Israeli sample as opposed to the Japanese one.

(2) The behaviors of the correlations (for instance, between intrinsic motivation and JE or HWI-TC) are different between the two groups of worker status, in each country *on its own*, such that (a) working students, in Israel, exhibit stronger links to the outcome variables (i.e., HWI-TC, HWI-TC, and JE) as opposed to non-student employees; (b) however, in most cases, these associations were not so different between said groups, in the Japanese sample.

(3) The behaviors of the correlations (for instance, between intrinsic motivation and JE or HWI-TC) are different between the two groups of worker status when *comparing* each country, such that (a) working students, in Israel, exhibit stronger links to the outcome variables as opposed their Japanese counterparts; (b) however, in most cases, these associations were not so different between non-student employees (in Israel vs. Japan).

(4) The only analysis in which points 1–3 above do not apply is when using intrinsic motivation to predict HWI-WI (again, in a moderated-moderation model). It suggests that intrinsic motivation’s impact on the increased effort at work changes based on neither worker status nor the country/culture.

These findings support our hypotheses H6–H9: (1) worker status does moderate the links between work motivation and the outcome variables (HWI-TC, HWI-TC, and JE), and (2) county/cultural differences can moderate said relationships as well. Still, more importantly, they work as a conditioning moderator on the previous moderation (i.e., moderated moderation) in all of the analyses done.

## Discussion

The aims of the current paper were (1) to shed light on the relationship between intrinsic/extrinsic motivation and HWI of time (HWI-TC) and effort (HWI-WI) and JE, (3) to assess convergent and discriminant properties of JE in relation to HWI-TC and HWI-WI, and (4) to gauge the moderation effects of both worker status (working students vs. non-student employees) and country/culture (Israel vs. Japan) on said relationships (point 1) in a moderated-moderation analysis type. Our research hypotheses were supported to a great extent. The findings are summarized in Table 7.

**TABLE 7:** Results of hypothesis testing.

**Hypotheses**	**Results**
H1: Intrinsic motivation positively associates with JE.	Supported (Table 6)
H2: Extrinsic motivation positively associates with JE.	Supported (Table 6)
H3: Intrinsic motivation positively associates with both HWI-TC and HWI-WI.	Supported (Tables 4, 5)
H4: Extrinsic motivation positively associates with both HWI-TC and HWI-WI.	Supported (Tables 4, 5)
H5a: JE positively associates with HWI-TC.	Supported (partial support, in Japan) (Table 2)
H5b: JE positively associates with HWI-WI.	Supported (Table 2)
H5c: JE has a stronger association with HWI-WI than with HWI-TC.	Supported (Table 2)
H6: Worker’s status moderates the relationship between intrinsic motivation and HWI- TC, HWI-WI, and JE, such that the relationship will be weaker for working students than for non-student employees.	Supported (Figures 2, 4)
H7: Worker’s status moderates the relationship between extrinsic motivation and HWI-TC, HWI-WI, and JE, such that the relationship will be weaker for working students than for non-student employees.	Supported (Figures 3, 5)
H8: Country differences condition the moderation of worker’s status on the relationship between intrinsic motivation and HWI-TC, HWI-WI, and JE, such that the effect of worker’s status suggested in H6 will be weaker for Japanese than for Israelis.	Supported (Figure 6)
H9: Country differences condition the moderation of worker’s status on the relationship between extrinsic motivation and HWI-TC, HWI-WI, and JE, such that the effect of worker’s status suggested in H7 will be weaker for Japanese than for Israelis.	Supported (Figure 7)

### Theoretical Implications

Our research adheres to the very few studies that have tested and validated Snir and Harpaz’s (2015) HWI conceptual model between its various predictors (i.e., intrinsic/extrinsic motivation) with regards to specific moderators (e.g., worker’s status and country/culture). Our findings supported the model (see Snir and Harpaz, 2015, p. 6) and contributed to its incremental validity. Apart from realizing parts of the model’s structure and processes, we have also shown that the moderation effects suggested in the model may be conditioned by other moderators as well (in our study, country/culture differences), leading to more need for further research.

Although it is not the main focus of the current research, we have established some convergent and discriminant validity relationship between JE and HWI. Specifically, JE has a high convergent validity with HWI-WI, yet low convergent-borderline-discriminant validity with HWI-TC, increasing the need for exploring these issues further.

We have provided more evidence as to the critical role of culture in differentiating model and relationship behaviors. Our findings regarding the between-country differences found in the moderating effects of workers’ status supported our hypotheses, suggesting that compared to Israeli workplaces, those in Japan, indeed, put much emphasis in loyalty and cohesion. Japanese working students show similar work behavior (i.e., JE and HWI) as non-student workers. Attitudes, norms, and behavioral codes accepted in a country X may be quite different in country Y, not only in the general society but at the workplace as well. Concerning the workers’ status, it seems plausible that employees’ differing perceptions of the work context may affect their “readiness” to translate a drive to work to an actual HWI of JE, alone or in conjunction with cultural perceptions as well.

Furthermore, our findings on between-country differences have important insights for research in organizational learning. Employees’ continuous learning is essential for organizations to be competitive in the current and future VUCA world. Therefore, an organization needs to provide employees with opportunities to learn and support, which enables them to manage their work–study conflict effectively. However, as suggested in the results of the Japanese sample, it may be possible that cultural norms restrain workers from dedicating their time to learning. In addition to the effects of organization-level human resource development climate (Chaudhary et al., 2012), we also need to consider the effects of national-level culture in the examination of organizational learning practices and their consequences.

### Practical Implications

If JE is an organizational goal toward which many workplaces strive, their respective managers may very well need to enhance employees’ work motivation (such as offering more rewards or challenge), thus increasing the employees’ propensity for translating that motivation into actual HWI or JE.

The moderation effects emphasize the need for smart and careful management in workplaces with international employees, as we notice how different Israel is from Japan, for example. Managers and even service-givers must pay attention to these cultural differences when doing work with or for an entity (e.g., country, organization, or group) from outside the providing side’s national boundaries.

Besides, the stronger associations between work motivation and JE or HWI in Israeli sample (see Figures 2–7) suggest that working students virtually actuate more of their working drives into the behavioral expressions of their drives to work, thus investing heavier in them. That may be so because working students are keener on proving themselves to the organization toward the end goal of being recruited as permanent employees (supported by the results in Israel, as opposed to Japan). Hence, those who have less occupational security are more likely to translate their drive to work into actual HWI and JE. Nevertheless, in today’s economy, in which “occupational sense of security” appears to be declining, it seems plausible that in the future the moderated association between motivation and HWI, found in our paper, will diminish in strength or even dissipate entirely. This argumentation finds support in recent publications (e.g., Neuner, 2013; Koene et al., 2014; Weil, 2014). Perhaps working students are also more susceptible to organizational incentives (i.e., intrinsic or extrinsic), as opposed to their non-student counterparts (i.e., “regular” employees).

On the other hand, Japanese workers showed relatively weak relationships between work motivation and JE or HWI. These findings suggest that the Japanese workplace norm restrains working students from putting much effort to study, and thus, they work long hours for managing impression or making up for their “violation” of the workplace norm. Such workplace derives from traditional Japanese culture which emphasizes loyalty and dedication to the employer (Blomberg, 1994), and even modern companies in Japan expect employees to dedicate most of their life to the organization, resulting in much overtime work of Japanese workers (Franklin, 2017; Pilla and Kuriansky, 2018; Mason, 2019). Therefore, to encourage employees’ continuous learning and associating organizational learning, managers in Japanese firms need to reconstruct the workplace norm such that working students will not feel guilty by studying outside of their organization.

### Limitations and Future Research Directions

While our study has strength in the newness of findings and the use of an international sample, we should mention its limitations. First, our data are cross-sectional and single sourced. It limits the generalizability of the research and does not let us see if the findings are stable across time. Although it may not be a major limitation, our research was not focused on a specific industry, sector, or type of workers (e.g., high-tech, low-tech, services, or marketing and sales). While this bolsters the external validity of the research, it limits the construct validity of the results.

In our model, we included only individual differences as predictors and only contextual elements as moderators. As such, we recommend using a mix of said variables, such as “place” in the model, as predictors and moderators, so as not to be limited to one direction of explanations. For Snir and Harpaz’s (2015) model of HWI (p. 6), we only validated a part of it but did not include HWI as a mediator, but only as an outcome. Thus, we recommend using the full model to shed light on its possible processes, beyond predictor–outcome relationships. In addition, we urge researchers to investigate and identify more potentially interesting and relevant moderators, as we showed in our model (i.e., country/culture differences).

To expand our understanding of cultural difference, we recommend replicating our study in other countries with cultural similarities or differences to the ones used in the research, to broaden the generalizability and validity of our findings. As we noted previously, In Hofstede’s use of the term, Japan is higher in power distance, masculinity, and long-term orientation than Israel. Thus, this study might reveal the moderating effects of both these cultural dimensions and the worker’s status. However, this study only includes two countries, which might limit the generalizability of the results. Therefore, we suggest scholars worldwide to not only replicate our research in other countries but to also consider other cultural dimensions to generalize and expand our findings. Furthermore, in future international comparative studies, researchers can explore why and how each country’s cultural and institutional components influence the differences that would exist between countries.

Concerning our findings regarding convergent and discriminant validity between JE and HWI, we also encourage more research to be done in order to provide a clearer picture regarding these validity issues we raised in the current study.

We suggest conducting longitudinal studies incorporating other potential moderator variables (such as work ethic and gender) or mediators (as previously mentioned) and further investigating processes—which we enumerated in the discussion section—as likely to connect work motivation to JE, HWI, and potential outcomes.

It is also safe to assume that the associations we discovered in the research would be dependent on which industry we focus on (e.g., high-tech, low-tech, marketing, or service), and as such, we would also suggest incorporating this element in future research.

Finally, we suggest that future research compare the effect of intrinsic and extrinsic motivation on various kinds of behavior using the same sample. Although this study is one of few studies that investigate the effect of both types of motivation in one study, it assumed that they result in similar attitude and behavior. As Ryan and Deci (2000a) argued, these two types of behavior can lead different kinds of behavior since their sources are different—that is, intrinsic motivation derives from one’s free choice, but extrinsic motivation is promoted by external controls. Therefore, future research can include various kinds of behavior in a model and explore whether these two types of motivation lead to a different behavior and why.

## Data Availability Statement

The datasets generated for this study are available on request to the corresponding author.

## Ethics Statement

The procedure of this study was approved by the Ethics Committee of Hosei University Graduate School of Career Studies. The committee approved that this study does not contain ethical flaws like leaking of private information and inhumane questions in the questionnaire. All subjects gave written informed consent regarding the purpose of research, that of data collection, and the privacy protection method. The current study was correlational, based on a survey, and not a manipulation on subjects. At the beginning of each questionnaire, we explained the general goal of the research. Informed consent was obtained from all individual participants included in the study. We ensured anonymity and discretion of the results and also ensured that the subjects know they could leave the participation at any time they choose.

## Author Contributions

All authors listed have made a substantial, direct and intellectual contribution to the work, and approved it for publication.

## Conflict of Interest

The authors declare that the research was conducted in the absence of any commercial or financial relationships that could be construed as a potential conflict of interest.
